# The Effect of Surface Modification of Aligned Poly-L-Lactic Acid Electrospun Fibers on Fiber Degradation and Neurite Extension

**DOI:** 10.1371/journal.pone.0136780

**Published:** 2015-09-04

**Authors:** Nicholas J. Schaub, Clémentine Le Beux, Jianjun Miao, Robert J. Linhardt, Johan G. Alauzun, Danielle Laurencin, Ryan J. Gilbert

**Affiliations:** 1 Center for Biotechnology and Interdisciplinary Studies, Rensselaer Polytechnic Institute, Troy, NY, 12180–3590, United States of America; 2 Department of Biomedical Engineering, Rensselaer Polytechnic Institute, Troy, NY, 12180–3590, United States of America; 3 Institut Charles Gerhardt de Montpellier, UMR 5253, CNRS-UM-ENSCM, Université de Montpellier, CC 1701, Place E. Bataillon, F-34095 Montpellier cedex 05, France; 4 Department of Chemical and Biological Engineering, Rensselaer Polytechnic Institute, 110 8^th^ Street, Troy, NY, 12180–3590, United States of America; 5 Department of Biology, Rensselaer Polytechnic Institute, 110 8^th^ Street, Troy, NY, 12180–3590, United States of America; 6 Department of Chemistry and Chemical Biology, Rensselaer Polytechnic Institute, Troy, NY, 12180–3590, United States of America; Michigan Technological University, UNITED STATES

## Abstract

The surface of aligned, electrospun poly-L-lactic acid (PLLA) fibers was chemically modified to determine if surface chemistry and hydrophilicity could improve neurite extension from chick dorsal root ganglia. Specifically, diethylenetriamine (DTA, for amine functionalization), 2-(2-aminoethoxy)ethanol (AEO, for alcohol functionalization), or GRGDS (cell adhesion peptide) were covalently attached to the surface of electrospun fibers. Water contact angle measurements revealed that surface modification of electrospun fibers significantly improved fiber hydrophilicity compared to unmodified fibers (p < 0.05). Scanning electron microscopy (SEM) of fibers revealed that surface modification changed fiber topography modestly, with DTA modified fibers displaying the roughest surface structure. Degradation of chemically modified fibers revealed no change in fiber diameter in any group over a period of seven days. Unexpectedly, neurites from chick DRG were longest on fibers without surface modification (1651 ± 488 μm) and fibers containing GRGDS (1560 ± 107 μm). Fibers modified with oxygen plasma (1240 ± 143 μm) or DTA (1118 ± 82 μm) produced shorter neurites than the GRGDS or unmodified fibers, but were not statistically shorter than unmodified and GRGDS modified fibers. Fibers modified with AEO (844 ± 151 μm) were significantly shorter than unmodified and GRGDS modified fibers (p<0.05). Based on these results, we conclude that fiber hydrophilic enhancement alone on electrospun PLLA fibers does not enhance neurite outgrowth. Further work must be conducted to better understand why neurite extension was not improved on more hydrophilic fibers, but the results presented here do not recommend hydrophilic surface modification for the purpose of improving neurite extension unless a bioactive ligand is used.

## Introduction

Autologous peripheral nerve grafts have been used to restore function after spinal cord injury (SCI) in animal models to recover limb function,[1] respiratory function,[2] and most recently bladder control.[3] However, strategies that harvest autologous nerve grafts damage the peripheral nervous system or risk rejection if the graft is allogeneic. Instead of harvesting peripheral nerve tissue, synthetic guidance channels have the potential to guide regenerating axons. Unfortunately, synthetic guidance channels do not consistently promote regeneration (as observed in peripheral nerve animal studies) at a faster rate than their autologous counterparts.[4] Thus, continued development of strategies to enhance the rate of nerve regeneration using synthetic guidance channels is required.

Many synthetic guidance approaches are studied in animal models of SCI.[5,6] One alternative approach to the autologous nerve graft is electrospinning, which is capable of creating aligned fibers with diameters on the nano to micro scale (reviewed by Lee and Livingston Arinzeh).[7] Electrospun fibers have the potential to direct axonal regeneration when fibers are highly aligned.[8–12] Additionally, therapeutic agents can be encapsulated in the polymer [13] for local, sustained delivery without compromising the fiber’s ability to guide axons.[14,15] Several recent studies have used electrospun fibers in animal models of SCI. The results from these studies reveal the ability of electrospun containing scaffolds to direct axonal regeneration[16,17] and astrocyte migration.[18] Some studies have also observed recovery of lost function when animals received electrospun fiber treatment in specific injury models more conducive to functional recovery (hemisection model vs. complete transection model).[19]

To realize the full potential of electrospun fibers and enable their translational use following SCI, different *in vitro* studies have modified electrospun fibers to increase the rate and length of neurite extension,[20–26] to improve astrocyte biocompatibility,[12,27] or improve myelin formation by oligodendrocytes.[28,29] *In vitro* studies attempting to optimize fiber geometry for enhanced neurite outgrowth have examined the effects of changing fiber alignment or diameter on neurite outgrowth from cell lines or explant models.[8,9,11,12,30–35] Additionally, some studies have attempted to improve cellular adhesion or neurite extension by adding extracellular matrix or growth factor proteins to the fibers. The addition of protein to the fibers was achieved by adding proteins to the electrospinning solution prior to electrospinning,[22–24,36,37] adsorbing the protein to the surface of the fiber,[38] or covalently attaching the protein to the fiber.[20,25,26]. Other studies have attempted to improve the hydrophilicity of electrospun scaffolds in order to improve neuronal cell adhesion and neurite extension by plasma treating the fibers.[39,40] Thus, post-fiber modification of fibers to improve fiber hydrophilicity or to incorporate a protein may enable more efficient regeneration within electrospun fiber channels following SCI.

In this study, we hypothesized that improvement of fiber hydrophilicity would increase the rate of neurite extension from chick DRG explants. We formed this hypothesis based on previous studies that show a correlation between improved cell viability and scaffold hydrophilicity,[41,42] with one study showing increased hydrophilicity and neuronal cell adhesion on fibers treated with oxygen plasma.[39] These studies are at odds with at least one study that found decreased neuronal viability on fibers with improved hydrophilicity following air plasma treatment.[40] However, there is overall little evidence on how the hydrophilic character of the fibers alters neurite extension. Since an improvement in hydrophilicity can be accomplished by using different surface functionalities, the discrepancies in previous studies could be attributed to differences in surface chemistry. Therefore, we modified electrospun fibers with different surface chemistries to understand how hydrophilicity changes neurite extension.

## Results and Discussion

### Surface Chemistry Stability Following Fiber Modification

Electrospun fibers have been modified previously to contain growth factors,[26] extracellular matrix components,[25] enzymes,[43] and surface plasma treatment,[41,44] but none of these studies evaluated the longevity of the surface modification. The longevity of the surface modification may be an important consideration for some applications, such as spinal cord injury where months of axonal extension are required in order to achieve recovery of lost function in rodents.[2,3] Therefore, we determined the presence of functional groups on the surface of electrospun, PLLA fibers at short time points after degradation in PBS (up to seven days) to understand how long the functional groups remained on the surface.

**Fig 1:**
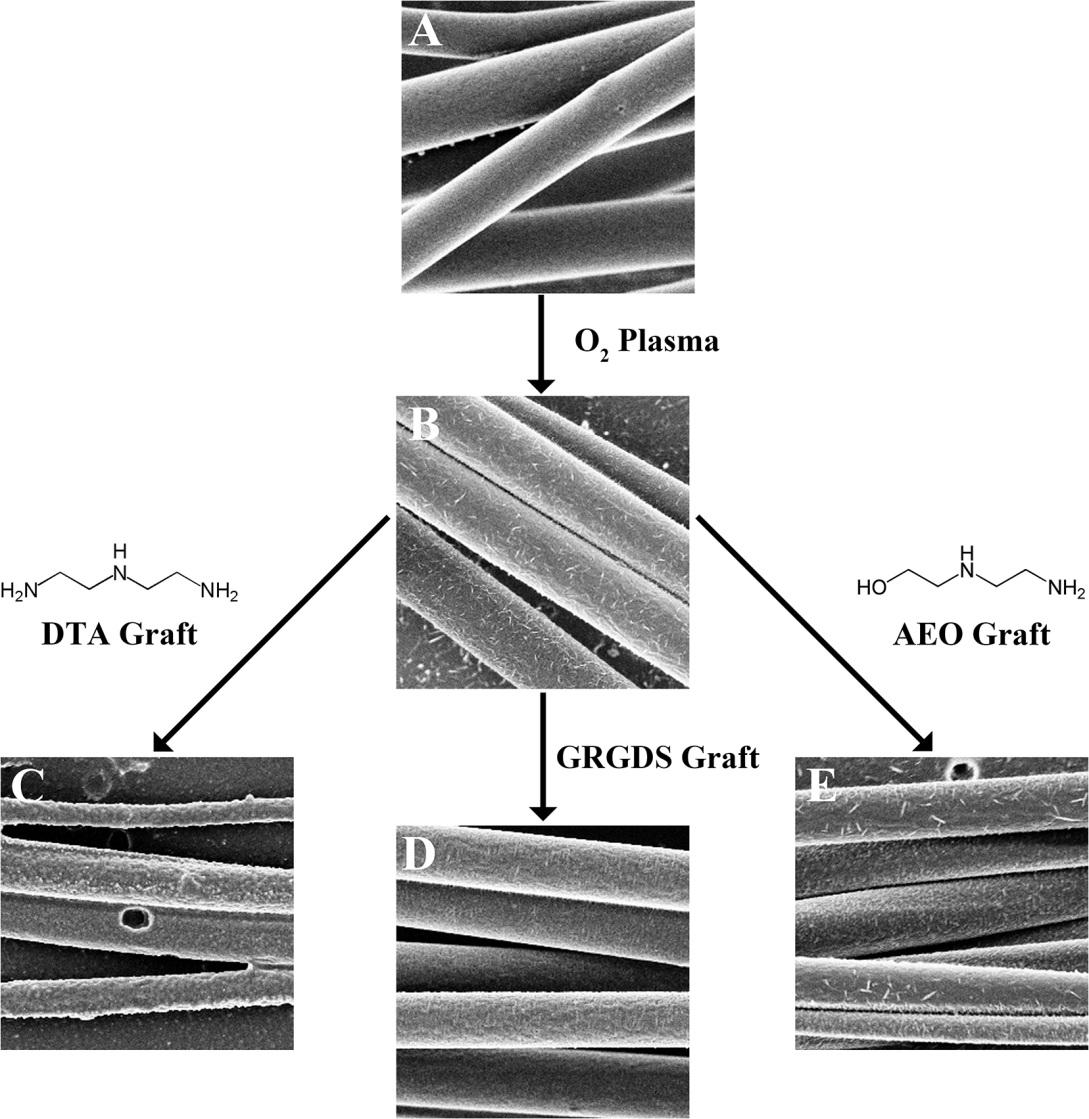
An outline of the reaction used in this study to modify the surface chemistry of electrospun fibers. Electrospun PLLA fibers from the same electrospinning trial are either unmodified (A) or treated with oxygen plasma (B). Then, some fiber groups were submerged in a solution containing EDC, NHS, and one of three ligands: DTA (C), GRGDS peptide (D), or AEO (E). Since the EDC/NHS reaction binds an amine group from one of the ligands (DTA, AEO, or GRGDS) to the fibers, the opposite end of the ligand will be exposed on the fiber surface.

All modified groups of electrospun, PLLA fibers were coupled to a ligand using an EDC/NHS reaction,[45] which was used previously to couple laminin to electrospun, PLLA fibers.[25] PLLA fibers were treated with oxygen plasma to place carboxylic groups on the surface of the fibers that served as functional sites for the peptide coupling reaction (Fig 1). Then, either diethylenetriamine (DTA), 2-(2-aminoethoxy)ethanol (AEO), or a GRGDS peptide was coupled on the surface of the fibers using the EDC/NHS combination. The EDC/NHS reaction couples a primary amine group to a carboxylic acid group, so the terminal amine group on the AEO will bind to the polymer leaving the terminal hydroxyl group free. The DTA modified groups will contain a free primary amine group, the other one forming a peptide bond with the PLLA. Finally, an amine group on either the terminal glycine or the arginine will link the GRGDS group to the polymer. The controls were unmodified PLLA fibers, and PLLA fibers exposed only to oxygen plasma. All fibers were then placed in PBS for 1, 2.5, or 7 days and analyzed using X-ray photoelectron spectroscopy (XPS).

**Fig 2:**
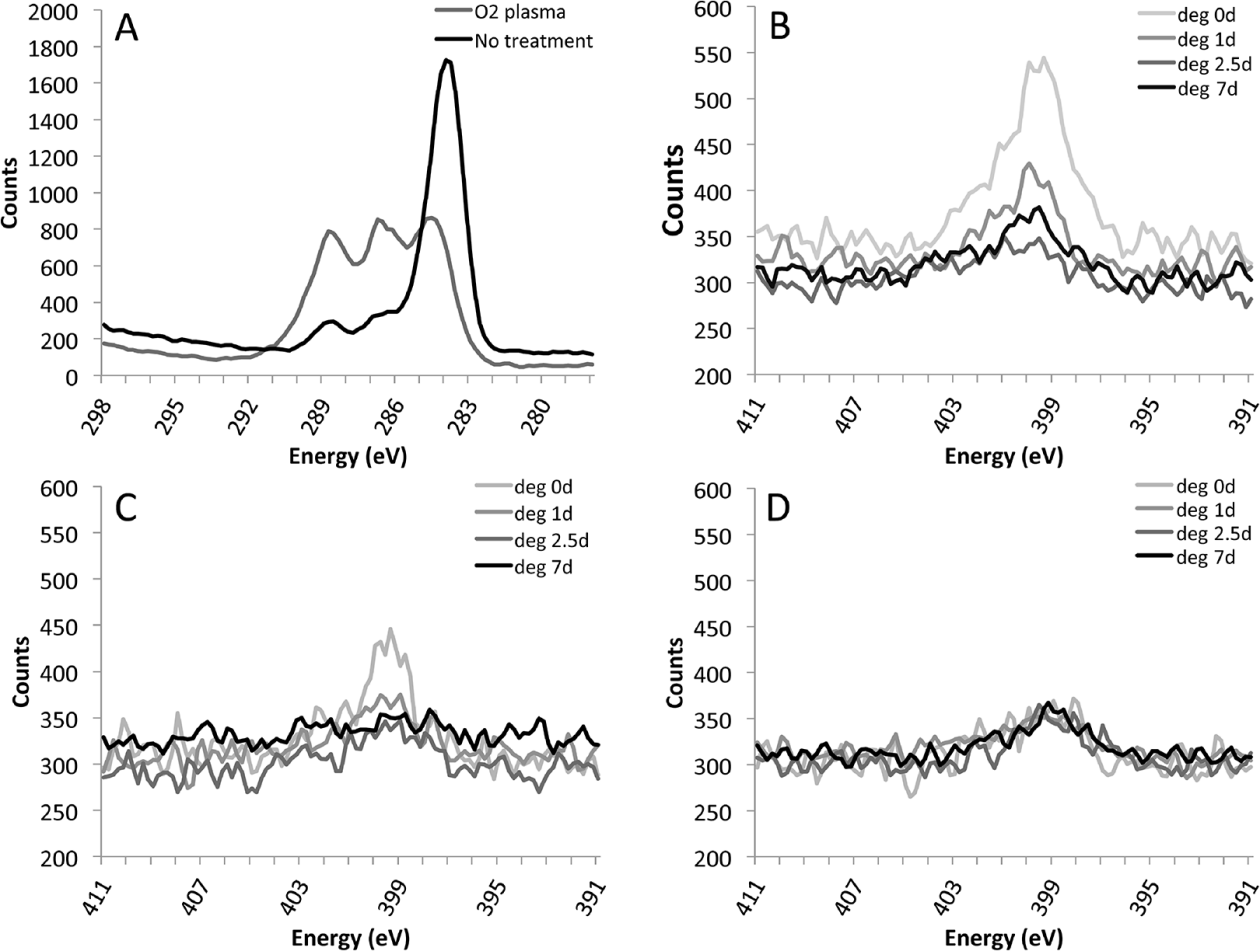
XPS of electrospun scaffolds to determine the presence of specific surface chemistries over time. First, the C1s region of the XPS data was evaluated to determine the change in surface chemistry before and after oxygen plasma treatment (A). The three peaks in (A) correspond to C-C and C-H bonds (283.4 and 284.8 eV), C-O bonds (286.8eV), and C = O bonds (289eV). Next, the nitrogen region of each scaffold was used to determine the presence of DTA (B), AEO (C), or GRGDS (D). The decrease in the nitrogen signal for DTA and AEO suggest surface erosion may be removing the surface chemistry. No decrease in signal is observed for the GRGDS peptide, suggesting GRGDS remains bound to the surface of the fibers throughout seven days of degradation.

We hypothesized that fiber surface modification would be transient since the covalently linked molecules contain hydrophilic species. The increased hydrophilic character of the surface would increase the rate of water diffusion into the fibers, and consequently the rate of degradation of the fibers.[46] In the C1s region of the XPS spectra of untreated and plasma treated PLLA fibers (Fig 2A), there were three peaks corresponding to C-C and C-H bonds (283.4 and 284.8 eV), C-O bonds (286.8 eV), and C = O bonds (289 eV). After oxygen plasma treatment, the relative proportion of these three types of bonds changed, with an increase in the relative number of C = O and C-O functional groups. The change in proportions of these functional groups following oxygen plasma treatment of PLLA fibers is in agreement with previous studies.[42,44] In the case where DTA (Fig 2B), AEO (Fig 2C), or GRGDS (Fig 2D) were covalently linked to the fiber surface, the nitrogen region within the XPS spectrum was used to confirm the presence of the grafted molecules on the fiber. In all cases, the appearance of a nitrogen signal at 399.6 eV reveals the presence of these species at the surface. The nitrogen peak observed in XPS analysis of fibers with DTA coupling immediately after the grafting reaction (Fig 2B, Day 0) was higher than the nitrogen peak from fibers with AEO treatment (Fig 2C, Day 0). The higher nitrogen signal for DTA compared to AEO at day 0 is likely due to the DTA molecule containing more nitrogen than AEO. After immersion of the fibers in PBS for defined periods of time, the nitrogen peak intensity decreased over time for the AEO and DTA grafted samples (Fig 2B and 2C), but there was no noticeable decrease in the XPS nitrogen peak for fibers grafted with the GRGDS peptide (Fig 2D). Based on these observations, we conclude that the amount of DTA and AEO present at the surface decreases over seven days to a nearly undetectable level, but the presence of GRGDS does not change significantly.

Very few studies have quantified electrospun fiber surface chemistry over time, and the observation that surface modifications may change over time may be an important factor in understanding the cell response. Klinkhammer *et al* performed XPS on electrospun fibers after degradation in water, but they did not assess if the chemical modifications they made to the fibers was lost over time.[21] Koh *et al* did a side by side comparison of different methods of placing laminin on the surface of electrospun, PLLA fibers.[25] Koh’s study compared electrospun, PLLA fibers that were covalently linked to laminin, had laminin physically adsorbed to the fibers, or a PLLA solution electrospun with laminin in it. Koh *et al* found that fibers electrospun with laminin in the electrospinning solution were best at achieving the longest neurite extension from PC12 neurons after five days in culture. While the authors of the study provided no explanation for the observed results, one potential explanation might be that the covalently linked or physically adsorbed laminin did not remain at the surface for the entirety of the culture period. Our results suggest that this may happen over a very short time period (days), but it may also be dependent on the molecule that is linked to the fibers since the GRGDS sequence did not decrease over time while the other molecules did. More work must be performed to understand how chemical modification to the surface of electrospun fibers changes over time. However, the results of this study lead us to conclude that there is a necessity to characterize the surface of modified fibers to determine whether the chemical modification remains at the surface of the fibers for the duration of the cell culture period.

### Electrospun Fiber Surface Structure and Fiber Diameter After Degradation

One important feature when considering the cell response to electrospun fiber scaffolds is the mean diameter of the fibers. Most cell types appear to have a unique response to micro-scale electrospun fibers compared to nano-scale electrospun fibers, and this is especially true for cells in the nervous system. Neurons tend to extend longer neurites on fibers with diameters a few microns in diameter compared to neurons cultured on fibers with nano-scale fiber diameters.[31,33,34,47,48] Therefore, it is important to understand whether surface modification of electrospun fibers increases the rate of surface degradation. If an increased rate of surface degradation took place, fiber diameter may decrease over time and cause a change in the cell response. The high surface area to volume ratio of electrospun fibers is frequently mentioned in the literature, suggesting surface modification might have a major impact on the degradation behavior of the fibers. While some work has been performed to understand how chemical modification of polymers holds up to degradation,[21] no study has determined whether surface erosion is a major issue for surface modified fibers.

**Fig 3:**
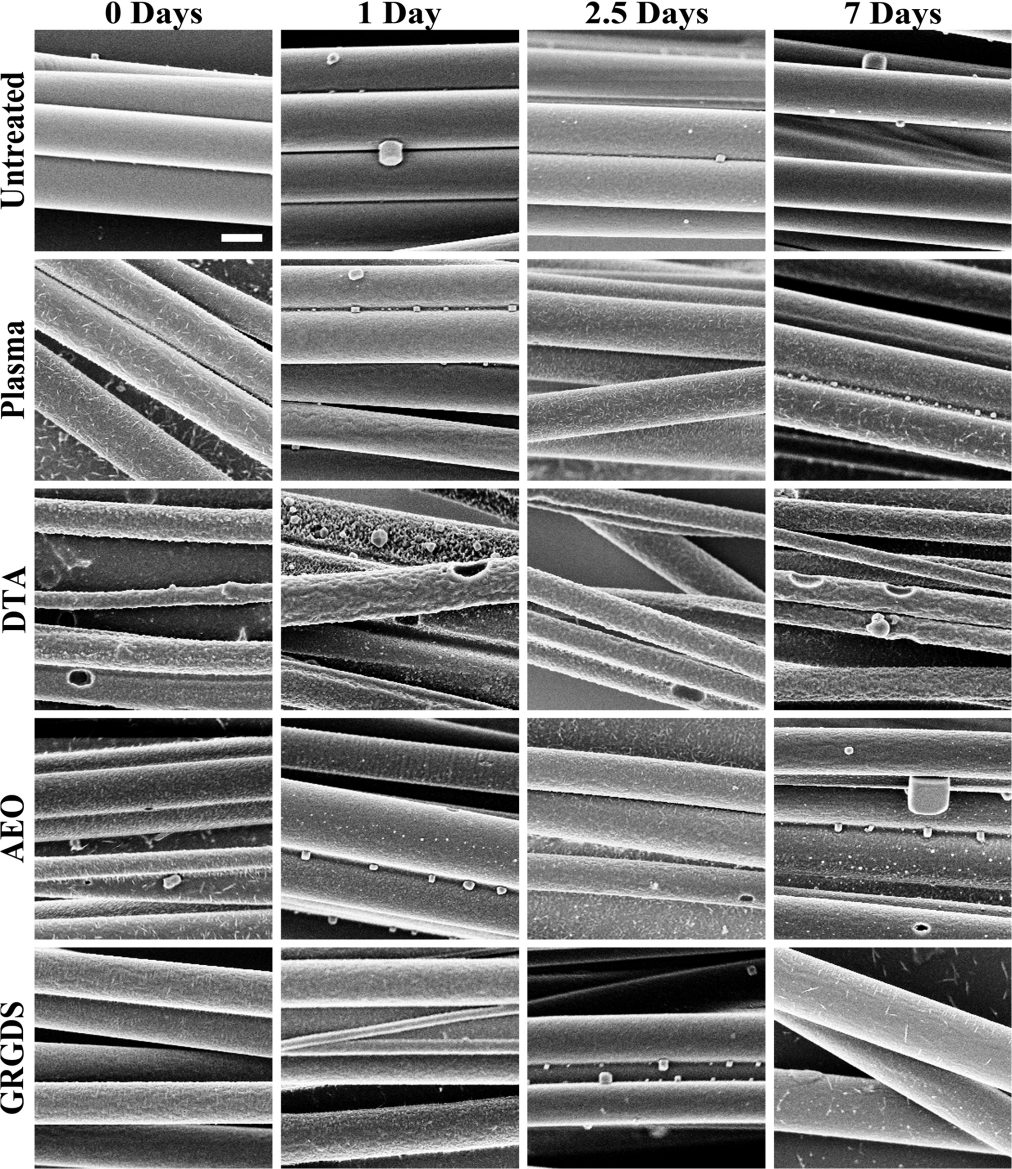
Surface morphology of electrospun fibers before chemical modification (Untreated row) or after different chemical modifications to the fibers. Fibers were imaged by SEM before degradation (Day 0) or after 1, 2.5, or 7 days in PBS. Untreated fibers were smooth, but plasma treated fibers or fibers modified with GRGDS or AEO showed a rougher surface. DTA fibers appeared even rougher, and contained pits in the surface of the fibers. No trends in fiber surface morphology were observed at any time point, suggesting differences in surface roughness were a result of the different chemical modifications. All images were captured at the same magnification, the scale bar (top right image) is 1 **μ**m.

Based on the XPS results that revealed a decrease in nitrogen signal at the surface of the AEO and DTA groups, we hypothesized that surface erosion would cause fiber diameter to decrease over time for all electrospun fiber scaffolds that underwent surface modification. To test this hypothesis, electrospun fibers with surface modifications (AEO, DTA, or GRGDS) were placed in PBS for 1, 2.5, and 7 days and then imaged using SEM. Control groups included unmodified fibers and fibers subjected to oxygen plasma alone. SEM images of each fiber group (Fig 3, each image is representative of three independent trials) revealed that the unmodified fibers had a smooth surface while all other groups appeared to have a rougher surface. Electrospun fiber scaffolds covalently linked to AEO or GRGDS appeared to have similar surface structure to electrospun scaffolds exposed to plasma alone. However, scaffolds covalently linked to DTA appeared much rougher than all other fibers and possessed large pits. The AEO fibers contained pits that were smaller and less frequent than the pits on the DTA fibers. Although fiber surface roughness between the control (smooth surface), DTA coupled fibers (rough surface with pits), and all other groups (AEO, GRGDS, and plasma treated fibers had a rough surface) appeared different at day 0 (before degradation), there did not appear to be a change in the surface structure over time in any scaffold. Since no apparent changes were observed in the surface structure of any fiber group over time, we conclude that plasma treatment and chemical modification are responsible for the changes in surface structure from one fiber group to another (presence of roughness and pit structure) and not due to hydrolysis. This observation also suggests that surface erosion does not play a major role in the degradation process of PLLA fibers over a short time period (seven days or less).

**Fig 4:**
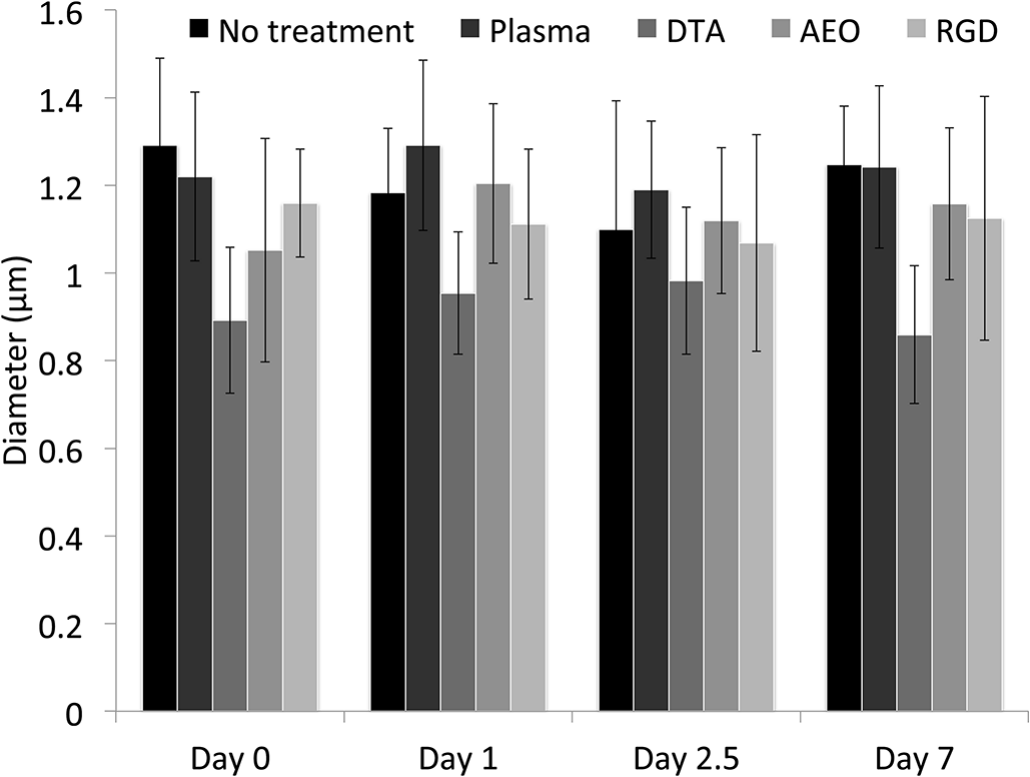
Analysis of fiber diameter after treatment or after 1, 2.5, or 7 days of degradation in PBS. No significant differences were observed between any groups at any time point, or with respect to time. The only trend observed was in the DTA fibers, which were consistently smaller than all other groups at every time point. These results suggest surface erosion of the fibers did not significantly alter fiber diameter after degradation over a 7-day period.

The suggestion that significant surface erosion of the modified fibers does not occur when exposed to PBS for a few days was further supported by a quantitative assessment of fiber diameter. In addition to fiber diameter, fiber alignment and fiber density were also quantified, but no statistical differences were observed for fiber diameter, alignment, or fiber density between any fiber groups at any time point (Fig 4, n = 3 independent samples for each time point). The only trend observed in the data was with respect to the DTA modified scaffolds, which always had fibers with smaller diameters than all groups at every time point, but this result was not statistically significant. It is possible that a few nanometers have eroded from the surface since the XPS data shows a decrease in nitrogen signal. Since we did not observe trends within a single group with respect to time, the variability of surface structure and diameter between groups led us to believe that the most significant change in surface structure and diameter was caused by the individual molecules (AEO, DTA, or GRGDS) during the EDC/NHS coupling reaction. Using both the qualitative assessment of fiber morphology and the quantitative assessment of fiber diameter, we can reject our original hypothesis that surface erosion of the functionalized fibers would cause a significant decrease in fiber diameter.

### Analysis of Fiber Hydrophilicity

Following XPS characterization, we wanted to determine if the different surface chemistries appreciably changed surface hydrophilicity. Furthermore, we wanted to determine whether the hydrophilic character of the fibers changed over time since the XPS data suggested that the surface chemistry of the modified fibers changed over time. Thus, we hypothesized that plasma treatment and surface modification would enhance the hydrophilicity of electrospun fibers. Oxygen plasma was used to covalently couple different molecules to the surface of electrospun fibers, likely increasing the hydrophilic character of the fibers before individual molecules were covalently linked to the fibers. Additionally, all molecules (AEO, DTA, and GRGDS) covalently linked the fiber surface contained hydrophilic groups (amine or alcohol), further increasing the hydrophilic character of the scaffolds. To assess the degree of hydrophilicity, the water contact angle was measured on three independently fabricated scaffolds for each surface modification group.

**Fig 5:**
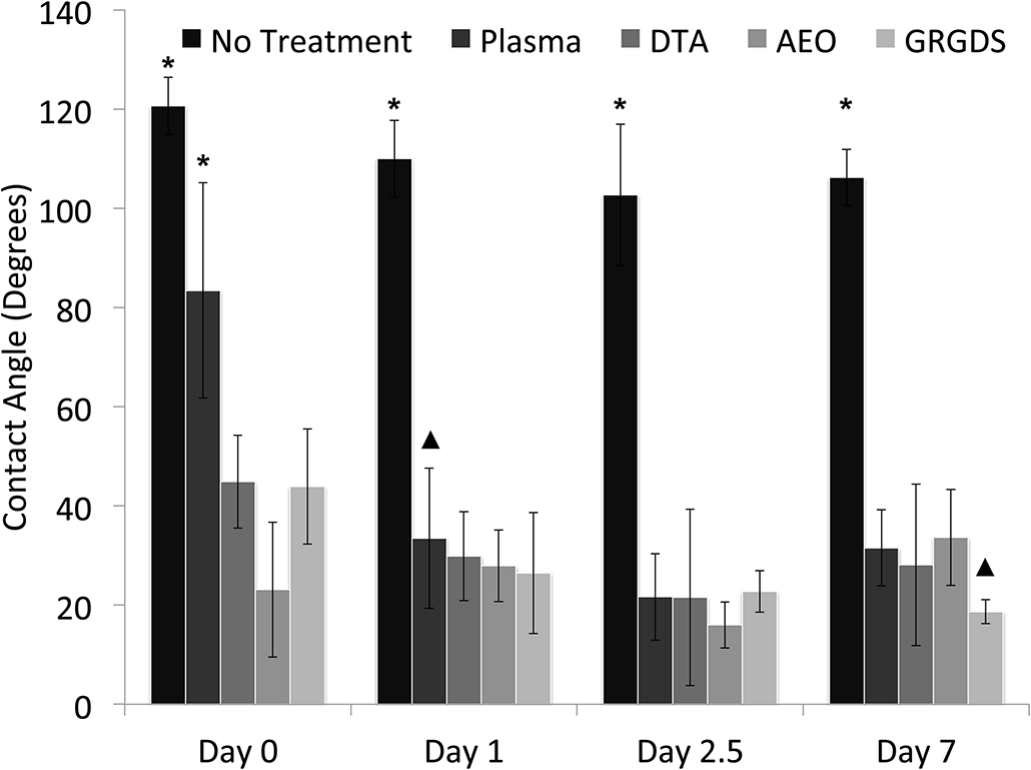
Measurement of water contact angles before degradation (Day 0) and after degraded in PBS for 1, 2.5, or 7 days. On Day 0, the No Treatment and Plasma groups are statistically significant from all other groups, including each other (*, p < 0.05). On every other day, only the No Treatment group is statistically significant from all other groups (*, p < 0.05). No group showed a significant change with respect to time, except for the Plasma treated group between Day 0 and Day 1 and the GRGDS group between Day 0 and Day 7 (▲, p < 0.05).

Fig 5 presents the water contact angle measurements on three independently fabricated scaffolds for each fiber scaffold with a different surface modification and the unmodified control (n = 3). As expected, the most hydrophobic group (i.e. largest water contact angle) was the unmodified PLLA fibers. Fibers treated with oxygen plasma had significantly lower water contact angle compared to the unmodified fibers (p < 0.05). Fibers with a surface modified with AEO, DTA, or GRGDS peptide had a smaller water contact angle than fibers that were untreated or plasma treated (p < 0.05). In addition to determining scaffold wettability immediately after chemical treatment (day 0), fibers were submerged in PBS to observe if surface hydrophilicity changed over time. The only significant decrease in water contact angle observed over time was on the plasma only group between day 0 (83.4° ± 21.7°) and day 1 (33.4° ± 14.1°, p < 0.05) and in the GRGDS group between day 0 (43.9° ± 11.6°) and Day 7 (18.7° ± 2.4°). Based on these results, we confirmed our hypothesis that the initial hydrophilicity of electrospun fibers was improved by altering the surface chemistry.

### Neurite Outgrowth from Chick DRG Cultured on Various PLLA Fiber Substrates

Electrospun fibers designed for nerve regeneration have typically been made of hydrophobic materials, such as PLLA [8,23,33,35,40,44] and polycaprolactone [37,49,50], but natural materials such as collagen [17,51] and laminin [36] have also been used. As presented in the introduction, there have been attempts to improve the hydrophilicity of electrospun fibers to improve neuronal adhesion and neurite extension with varying results. In this study, two small molecules (DTA and AEO) were selected to better understand whether the hydrophilic character of electrospun fibers with different surface chemistries could improve neurite extension. In addition to these two molecules, a bioactive ligand known to interact with integrins was also used for comparison (GRGDS).[52,53] Using these different surface chemistries, we attempted to understand how surface chemistry and hydrophilicity might be used to improve neurite extension on aligned, electrospun PLLA fibers.

We hypothesized that neurite extension from DRG would be greater on PLLA fibers with a modified surface chemistry (more hydrophilic) than the unmodified control (more hydrophobic). Additionally, we hypothesized that since the GRGDS peptide would induce the greatest amount of neurite extension due to its bioactivity. Chick DRG were cultured on the different substrates in the absence of serum, since serum proteins may adsorb to the surface of the fibers and mask the fiber chemistry.

**Fig 6:**
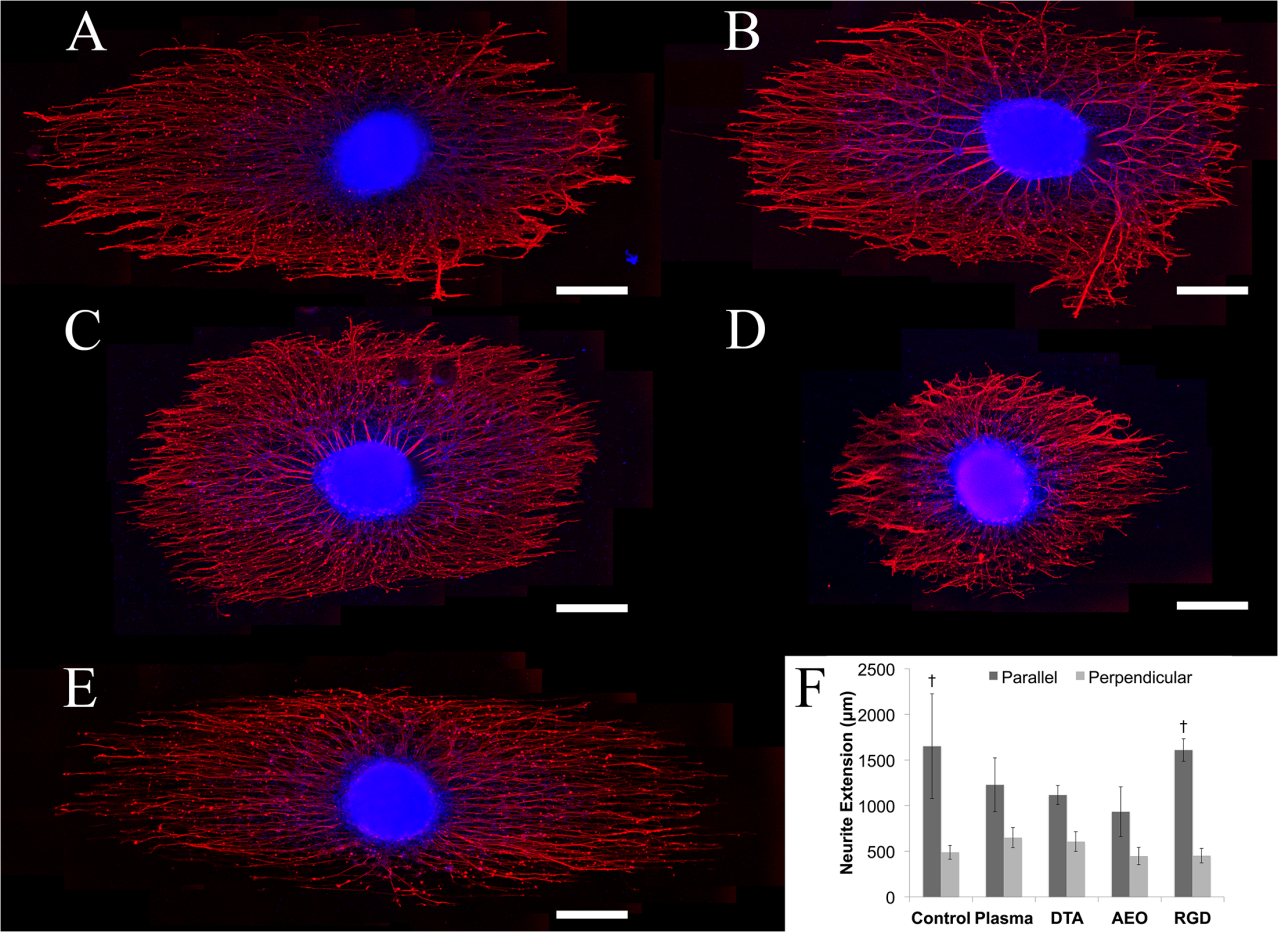
Neurite extension from chick DRG on electrospun PLLA fibers (A) and after plasma treatment (B) or after modification with DTA (C), AEO (D), or GRGDS (E). Measurement of maximum neurite extension (F, n = 3) revealed that the maximum neurite extension occurred on untreated fibers and fibers modified with GRGDS, and the untreated fibers and fibers modified with GRGDS were statistically significant from the fibers modified with AEO (†, p < 0.05). This data suggests no advantage to improving electrospun fiber hydrophilicity since all groups are more hydrophilic than the untreated control, but maximum neurite extension does not improve on any group compared to the control. All scale bars are 400 **μ**m.

Unexpectedly, neurites from DRG cultured on the control group (Fig 6A, 1651 ± 488 **μ**m) were longer than neurites from DRG cultured on plasma treated fibers (Fig 6B, 1240 ± 143 **μ**m) or DTA fibers (Fig 6C, 1118 ± 82 **μ**m), but these results were not statistically significant. However, neurites from DRG cultured on AEO fibers (Fig 6D, 844 ± 151 **μ**m) were significantly shorter than neurites from DRG on control fibers (p<0.05). Neurites from DRG cultured on GRGDS modified fibers (Fig 6E, 1560 ± 107 **μ**m) were about the same length as neurites on control fibers, and were statistically longer than neurites from DRG cultured on AEO fibers (p<0.05). Thus, neurites from DRG cultured on unmodified, control fibers and GRGDS modified fibers were the longest, followed by neurites from DRG on plasma and DTA fibers, and the neurites from DRG cultured on AEO modified fibers were the shortest. Neurites from DRG cultured on plasma treated fibers showed a significant increase in the maximum perpendicular neurite extension compared to the neurites from AEO group (p < 0.05), but no other groups showed statistical differences (Fig 6F). The similarity in neurite extension between the unmodified fibers and fibers containing GRGDS peptide (both parallel and perpendicular to fiber orientation) makes us conclude that there is no clear advantage to covalently coupling an RGD peptide in terms of enhancing the length of neurite outgrowth from DRG at the concentration applied to the fibers. Additionally, the reduction in maximum neurite extension along the fibers and increased perpendicular neurite extension led us to conclude that plasma, DTA, or AEO modified fibers do not improve maximum neurite extension and was detrimental to neurite extension in the case of AEO modification. Based on these results, we find no improvement to neurite extension solely based on hydrophilic character.

At least one study has demonstrated that an improvement in material hydrophilicity would improve neuronal adhesion on PLLA,[39] but other studies demonstrated how electrospun fiber hydrophilicity generally leads to less adhesion in primary neuronal cells.[40,54] No study has determined how electrospun fiber hydrophilicity affects neurite extension, so we applied different chemistries to the fiber surface to determine how fiber hydrophilicity and surface chemistry affected neurite extension. All of the chemically modified groups showed a significant improvement in hydrophilicity, but none of the groups revealed an increase in neurite extension when chick DRG were cultured on the modified scaffolds. The results of our experiment are in agreement with another study that linked GRGDS to the surface of electrospun fibers. Klinkhammer *et al* found that addition of the GRGDS peptide to electrospun polycaprolactone (PCL) fibers marginally improved neurite extension compared to plain electrospun PCL fibers, but the results were not statistically significant.[21] Klinkhammer’s study used embryonic day 10 chick DRG, similar to our use of our day 9 chick DRG, but Klinkhammer used poly-ethylene glycol (PEG) to link the GRGDS peptide to the fibers. The results of Kinkhammer’s study and ours are similar since PEG modification alone increased the hydrophilicity of electrospun scaffolds but decreased neurite extension from chick DRG, similar to the observation in our study that plasma treatment alone increased the scaffolds hydrophilicity but decreased neurite extension. However, in both Klinkhammer’s study and this study, the addition of GRGDS to the fibers resulted in a similar amount of neurite extension as the unmodified control. The agreement between our results and Klinkhammer’s results gives us additional confidence in our conclusion that hydrophilicity does not improve neurite extension.

One important difference between the study by Klinkhammer *et al*.[21] and the present study is the use of culture media containing serum. In this study, serum was not used in order to reduce the amount of protein adsorbing to the fiber surface. This was done so that neurite extension on the scaffolds could be attributed to the interaction of the neuronal growth cone with the different surface modifications rather than the proteins adsorbed to the surface of the fibers. Previous studies have demonstrated that depleting serum containing media of certain proteins, such as fibronectin,[55] can prevent neurite extension, but other studies have demonstrated that neurites will grow on electrospun fibers in the absence protein adsorption from serum containing media.[8,40] Since we did not use serum containing media, few proteins could adsorb to the surface and the results can be directly attributed to the surface modifications. In contrast to the present study, Klinkhammer used serum containing culture media, and this may explain the marginal, but not significant, increase in neurite extension from DRG cultured on fibers containing GRGDS compared to the unmodified control. Since we used media without serum, thus preventing protein adhesion, it is possible to conclude that the chemical modifications used in this study were not directly beneficial to neurite extension. However, future studies will determine if these chemical modifications are beneficial to neurite extension by promoting protein adsorption.

One potential alternative explanation for our neurite extension result is the differences in surface roughness (i.e. nanotopography) of electrospun fibers between fiber groups. We recently reported a method of controlling the nanotopography of individual fibers by phase separation, and fibers with a rough nanotopography restricted RAW 264.7 cell elongation compared to fibers with a smooth nanotopography.[56] Even though RAW 264.7 cells are similar to macrophages, the results of that study demonstrate that the surface roughness of individual fibers alters cell spreading, and therefore surface roughness may alter neurite extension. However, an analysis of fiber surface morphology in comparison to maximum neurite extension suggests that fiber surface morphology likely did not play a significant role in our results. There is a similarity in the surface morphology of the plasma, AEO, and GRGDS grafted fibers, which appeared rougher than the smooth surface of the unmodified fibers (Fig 4). In spite of the similar surface morphology of the electrospun fibers, the GRGDS group and the AEO group were statistically significant form each other. Additionally, the DTA modified fibers appeared to have a much rougher surface that also contained pits, but the DTA fibers appeared to perform similarly to the plasma treated fibers. These conflicting results suggest that surface roughness did not significantly alter maximum neurite extension when there was similar hydrophilicity, and we conclude that it was the initial surface chemistry rather than surface roughness or hydrophilicity that altered neurite extension. Also, because the hydrophobic electrospun fibers had different surface chemistry, water contact angle, and surface roughness, it is possible that any combination of these three factors contributed to the similar neurite extension observed between the hydrophobic unmodified fibers and the GRGDS modified fibers. This observation still does not negate our conclusion that initial hydrophilicity does not improve neurite extension.

One variable unaccounted for in this study is the rate of neurite extension. Bockelmann *et al*. found that electrospun fibers containing GRGDS and a mixture of PCL and NCO-poly(ethylene glycol)-star-poly(propylene glycol) (sPEG) caused neurites from day 10 embryonic chicken DRG to extend at a faster rate than the same fibers without the GRGDS sequence.[19] However, Bockelmann’s study did not evaluate total neurite extension, as Klinkhammer *et al*. did.[20] Therefore, it may be possible that neurites may be limited to a maximum length, but the addition of GRGDS may increase the rate at which neurites are able to reach this maximum. Bockelmann’s study also reports that at a short time point (24 hours) the maximum neurite extension of DRG on electrospun fibers containing GRGDS is greater than the maximum neurite extension of DRG on electrospun fibers without GRGDS. However, maximum neurite extension from DRG cultured on PCL fibers compared to PCL/sPEG and GRGDS is not statistically significant, even though the rate of neurite extension is different between these two groups. The results from Bockelmann’s study suggest that the rate of neurite extension may improve maximum neurite extension, but it is currently unclear in the literature how maximum neurite extension and the rate of neurite extension are related. Since the purpose of our study was not to elucidate how maximum neurite extension and the neurite extension rate are related to each other, we chose to evaluate maximum neurite extension since this is the most common metric used in the literature to evaluate neurite extension on electrospun fibers.

Another potential explanation for the results we observed for the GRGDS modified fibers could be the concentration of GRGDS used. Schense *et al* demonstrated that a high level of RGD incorporated into a fibrin gel decreased neurite extension from embryonic day 8 chick DRG, while a low concentration of RGD in the fibrin gel promoted neurite extension from DRG.[57] Thus, it is possible that the concentration used in our study may have been too high to promote neurite extension. It is difficult to compare the concentration on the surface of our fibers to that of Klinkhammer’s study since Klinkhammer added the RGD to the electrospinning solution, and did not evaluate the amount of RGD on the surface of the fibers. Thus, it is possible that the marginal increase in neurite extension that Klinkhammer observed on GRGDS modified fibers could be caused by a smaller amount of GRGDS present at the surface of the fibers. The 20 μg/mL of GRGDS used to covalently link to the electrospun fibers was chosen for this study based on standard concentrations (1 **μ**g/mL-50 **μ**g/mL) commonly used to coat elecrospun surfaces with protein.[21,25,34,58–62] Since the purpose of this study was not to determine how GRGDS alters neurite extension, only one concentration was used. However, based on how the GRGDS group performed in comparison to the other experimental groups, a study to optimize GRGDS surface concentration may be warranted.

## Conclusions

Electrospun fibers are promising biomaterials for the construction of an artificial nerve graft. In this study, we found that surface modification of electrospun fibers improve scaffold hydrophilicity, but no scaffold performed better than the unmodified control scaffolds when analyzing total neurite extension from DRG. In the case of AEO and DTA modifications, we observed a rapid decrease in nitrogen signal in the XPS data that suggested that the AEO and DTA were being removed from the surface. The results of this study lead us to conclude that a characterization of electrospun scaffolds after surface modification is critical to determine the stability of the modification. Additionally, electrospun scaffold initial hydrophilicity does not improve neurite extension. Future work will focus on modifying the surface of the electrospun scaffolds using bioactive ligands rather than non-specific chemistries.

## Materials and Methods

### Creation of Electrospun Poly-L-Lactic Acid (PLLA) Fibers

All materials were purchased from Sigma-Aldrich (St. Louis, MO) unless stated otherwise. Electrospinning was performed using previously published methods.[8,14,33,56,63] Briefly, poly-L-lactic acid (NatureWorks 6201D, Minnetonka, MN) films were prepared by air casting a 4% PLLA solution (wt/wt) dissolved in a 1:1 (v/v) mixture of chloroform and dichloromethane onto 15 mm circular glass coverslips (Propper Manufacturing, Long Island City, NY). Then, 12% (wt/wt) PLLA dissolved in 1,1,1,6,6,6-hexafluoroisopropanol (HFP) was electrospun onto PLLA coated glass coverslips fixed to a rotating aluminum disk for 20 min using the following conditions: rotating speed of 1000 rpm, voltage of 15 kV, 5.5 cm collection distance, humidity of 30–33%, and 2 ml/h syringe pump flow rate. Three independently fabricated electrospun fiber groups were generated (n = 3), and all subsequent experiments used fibers from all three independently fabricated fibers.

### Fiber Surface Treatment with Oxygen Plasma

Oxygen plasma treatment was carried out on the electrospun fiber scaffolds using a PlasmaTherm 73 system (St. Petersburg, FL). The plasma discharge was created at a radio frequency of 100 W, and the PLLA fibers were exposed to the oxygen plasma for 30 s. The plasma treatment was conducted in triplicate for each group. After plasma treatment, the edges of the fiber samples were coated with polymethylsiloxane (Dow Corning, Midland, MI) to prevent the fibers and film from lifting off the surface of the glass during the surface modification procedure. To accomplish this, a small amount of polydimethylsiloxane was placed on a an 18 mm glass coverslip (Propper Manufacturing, Long Island City, NY) and the fibers contained on the 15 mm glass coverslip were placed face up in the center of the 18 mm glass coverslip. The 15 mm glass coverslip was then pressed down until the PDMS began to envelop the edges of the glass coverslip.

### Covalent Attachment of Chemicals to Electrospun Fiber Surface

After plasma treatment, the surface of the PLLA fibers was further modified chemically by using 0.5 mM of 1-ethyl-3-[3-dimethylaminopropyl)carbodiimide (EDC, Alfa Aesar, Ward Hill, MA) and 0.5 mM N-hydroxysuccinimide (NHS, Alfa Aesar) as coupling agents, and either diethylenetriamine (DTA, Alfa Aesar, Ward Hill, MA), 2-(2-aminoethoxy)ethanol (AEO, Alfa Aesar, Ward Hill, MA), or a GRGDS sequence as functionalizing molecules (Fig 1). The total volume of solution placed onto each electrospinning fiber sample was 5 ml. The grafting solutions were prepared using ultrapure water, and the reagents were added to create final concentrations of 0.5 M for DTA and AEO or 20 **μ**g/ml for the GRGDS. Fibers were placed in the reaction solution and gently mixed for two days under ambient conditions. The fibers were then washed successively with ultrapure water and ethanol by dipping them six times in each medium for 1 second each before a final immersion in ethanol for 5 hours. The successive dipping in each medium followed by the 5-hour immersion in ethanol was done to remove any unlinked chemicals. The fibers were then dried overnight in a vacuum desiccator at room temperature. This covalent linking was performed three times on independently fabricated samples.

### X-ray Photoelectron Spectroscopy

To verify the presence of different chemical species at the fiber surface following chemical modification, the chemical composition of the fiber surface was characterized by X-ray photoelectron spectroscopy (XPS) using a PHI 5000 VersaProbe (ULVAC-PHI, Inc). The surface signals of carbon, nitrogen and oxygen were analyzed on each experimental group.

### Degradation of PLLA fibers

By making the fibers more hydrophilic through covalent linkage of hydrophilic chemicals, we hypothesized that the fibers would degrade more quickly than without surface treatment. To assess fiber degradation, dry PLLA fibers were sterilized with a 70% ethanol (in ultrapure water) solution and placed in a 12-well plate with 1 ml of calcium and magnesium free phosphate buffered saline (PBS, Invitrogen, Life Technologies, Carlsbad, CA). After 1, 2.5, or 7 days of degradation, the PBS was removed. Next, degraded samples were dried in a vacuum desiccator before visualization of fibers using scanning electron microscopy. Three independently fabricated electrospun fiber scaffolds were degraded for analysis (n = 3).

### Measuring Fiber Hydrophilicity Using Water Contact Angle Measurement

Water contact angle analysis was performed to compare the hydrophilicity between the different fiber groups. Water contact angles were measured by the static sessile drop method under ambient conditions using a NRL C-A goniometer (Rame-hart, Inc., Mountain Lake, NJ). Water contact angle was measured along the length of the fibers, meaning the fibers were perpendicular to the direction the camera was facing. The measurements were repeated three times on independently fabricated samples (n = 3).

### Examining Fiber Surface Structure using Scanning Electron Microscopy

Fibers were first sputter coated with a thin layer of platinum (Denton Desk IV, Moorestown, NJ) in preparation for scanning electron microscopy (SEM). The edges of the fibers were then fixed onto the SEM sample holder using carbon tape. SEM images were taken using a Carl Zeiss Supra55 SEM (Thornwood, NY) at an accelerating voltage of 2 kV. Images were taken at 5000x and 15000x magnification.

### Measurement of Fiber Alignment, Density, and Diameter

To ensure that differences in cellular behavior between fiber groups were not attributable to changes in fiber physical properties, fiber alignment, diameters, and densities were characterized from SEM images. Average fiber diameter was characterized by measuring the length of a line drawn perpendicular to the length of ten individual fibers per image using ImageJ (National Institutes of Health, Bethesda, MD). Then, fiber alignment was determined by measuring the angle of a line drawn parallel to ten individual fibers per image. Finally, fiber density was determined using a line perpendicular to the orientation of the fibers. The number of fibers crossing the line was counted in order to determine the number of fibers per unit of length. Three independently fabricated samples were imaged, and the mean of each group was used for statistical analysis (n = 3).

### Culture of Chick Dorsal Root Ganglia (DRG) on Electrospun Fiber Scaffolds

Isolation of dorsal root ganglia (DRG) from chicken embryos was approved by the IACUC at Rensselaer Polytechnic Institute. DRG from three different E9 (embryonic stage) chicks were isolated from each side of the spinal cord in the lower thoracic region. Five different aligned, electrospun PLLA scaffold groups were used for DRG culture: 1) unmodified fibers without surface treatment (PLLA fibers), 2) plasma treated fibers (p-PLLA fibers), 3) DTA modified fibers (amine fibers), 4) AEO modified fibers (alcohol fibers), and 5) GRGDS modified fibers (RGD fibers). Five DRG were isolated from each chick (one for each scaffold). Thus, DRG from three different chick were cultured on three independently fabricated electrospun fibers in each group, giving three biological replicates with three fiber replicates for each electrospun fiber group (n = 3 DRG for each fiber group). DRG culture medium consisted of Neurobasal medium containing 0.5 mM L-glutamine, 1% penicillin/streptomycin, and 2% B-27 serum (all medium and additives were purchased from Invitrogen, Life Technologies, Carlsbad, CA). Just before adding the DRG to the fibers, a cloning cylinder (Sigma Aldrich, St. Louis, MO) was filled with 100 **μ**l of DRG cultured medium, and DRG were placed in the Neurobasal medium in the cylinder to ensure the DRG attached to the scaffold. After allowing the DRG to adhere to the electrospun scaffold for 12 hours in a 12-well plate in an incubator at 37°C and 5% CO_2_, the cloning cylinder was removed and additional media was added to a final volume of 1 ml and nerve growth factor (NGF, Calbiochem, La Jolla, CA) was added to a final concentration of 50 ng/ml. The DRG were then cultured on the fibers for 5 days in the same tissue culture incubator.

### Assessment of Neurite Outgrowth from DRG Cultured on Electrospun Fiber Scaffolds

After five days in culture, 1 ml of room temperature PBS containing 8% (w/v) paraformaldehyde (Electron Microscopy Sciences, Hatfield, PA) was added to the culture solution for a final concentration of 4% paraformaldehyde. After 30 min, the paraformaldehyde was removed and washed three times with PBS before a PBS blocking solution containing 10% fetal bovine serum (FBS, Invitrogen, Life Technologies, Carlsbad, CA) and 0.1% Triton X-100 was added to the cells and incubated at 4°C. After 12 h, the blocking solution was removed and a PBS solution containing 2% of FBS, 0.1% Triton X-100, and RT-97 antibody to label neurofilament (1:100, Developmental Studies Hybridoma Bank, Iowa City, IA) was added to the DRG cultures. After 8 h, the solution was removed, and the DRG were washed three times with PBS. Then, a PBS solution containing 2% of FBS, 0.1% Triton, and donkey anti-mouse antibody conjugated with Alexa-594 (1:1000 dilution, Sigma-Aldrich, St. Louis, MO) was added to the DRG cultures and incubated for 1 h. Finally, a solution of DAPI (1:1000, Sigma-Aldrich, St. Louis, MO) was added for 15 min before washing the cells three times with PBS. The cells were imaged with an Olympus DSU spinning disc confocal microscope (Center Valley, PA).

### Measuring the Extent of Neurite Outgrowth on Electrospun Fiber Scaffolds

The ten longest neurites from each side of the DRG were measured using ImageJ. Since each DRG culture was performed in triplicate, 60 neurite measurements were made for each group. The average of the longest neurites from each DRG were used for statistical analysis (n = 3 DRG). In addition to measuring the longest neurites, the maximum neurite growth perpendicular to the direction of the fibers was measured on each side of the DRG. The average of the two perpendicular neurite extension values were used for statistical analysis (n = 3 DRG).

### Statistical Analysis

Statistical analysis was made using JMP software (version 10). All groups were analyzed using a one way ANOVA followed by a Tukey-Kramer multiple comparisons test to determine differences between individual groups. Groups were considered statistically significant if p < 0.05. All data is represented in graphs and the text using the mean and standard deviation for all replicates for a particular electrospun scaffold group.
